# Ethical perceptions towards real-world use of companion robots with older people and people with dementia: survey opinions among younger adults

**DOI:** 10.1186/s12877-020-01641-5

**Published:** 2020-07-14

**Authors:** Hannah L. Bradwell, Rhona Winnington, Serge Thill, Ray B. Jones

**Affiliations:** 1 Center for Health Technology, University of Plymouth, Plymouth, Devon UK; 2 Department of Nursing, Auckland University of Technology, 90 Akoranga Drive, Auckland, New Zealand; 3 Donders Centre for Cognition, Radboud University, Nijmegen, 6525 HR The Netherlands

**Keywords:** Robot ethics, Machine ethics, Companion robots, Social robots, Older people, Aged care, Health and social care, Stakeholders, Gerontology

## Abstract

**Background:**

Use of companion robots may reduce older people’s depression, loneliness and agitation. This benefit has to be contrasted against possible ethical concerns raised by philosophers in the field around issues such as deceit, infantilisation, reduced human contact and accountability. Research directly assessing prevalence of such concerns among relevant stakeholders, however, remains limited, even though their views clearly have relevance in the debate. For example, any discrepancies between ethicists and stakeholders might in itself be a relevant ethical consideration while concerns perceived by stakeholders might identify immediate barriers to successful implementation.

**Methods:**

We surveyed 67 younger adults after they had live interactions with companion robot pets while attending an exhibition on intimacy, including the context of intimacy for older people. We asked about their perceptions of ethical issues. Participants generally had older family members, some with dementia.

**Results:**

Most participants (40/67, 60%) reported having no ethical concerns towards companion robot use when surveyed with an open question. Twenty (30%) had some concern, the most common being reduced human contact (10%), followed by deception (6%). However, when choosing from a list, the issue perceived as most concerning was equality of access to devices based on socioeconomic factors (m = 4.72 on a scale 1–7), exceeding more commonly hypothesized issues such as infantilising (m = 3.45), and deception (m = 3.44). The lowest-scoring issues were potential for injury or harm (m = 2.38) and privacy concerns (m = 2.17). Over half (39/67 (58%)) would have bought a device for an older relative. Cost was a common reason for choosing not to purchase a device.

**Conclusions:**

Although a relatively small study, we demonstrated discrepancies between ethical concerns raised in the philosophical literature and those likely to make the decision to buy a companion robot. Such discrepancies, between philosophers and ‘end-users’ in care of older people, and in methods of ascertainment, are worthy of further empirical research and discussion. Our participants were more concerned about economic issues and equality of access, an important consideration for those involved with care of older people. On the other hand the concerns proposed by ethicists seem unlikely to be a barrier to use of companion robots.

## Background

Robotics may provide a technological aid in meeting the increasing demand on health and social care [1], caused in part by increasing life expectancy [1–3], as human function deteriorates with age [4, 5]. Companion robots such as robot pets designed congruent with animal aesthetics and behaviours, have particular potential in aged care [6, 7]. The most well researched example is Paro, the robot seal [8]. Research has suggested numerous benefits of interacting with Paro, including reduced agitation and depression in dementia [9, 10], a more adaptive stress response [11], reduced care provider burden [11], and significantly improved affect and communication between dementia patients and day care staff [12]. Furthermore, Paro may reduce psychoactive and analgesic medication use [13], and even decrease blood pressure [14]. Alternatives to Paro include, amongst others, Miro, Pleo, and the Joy for All devices, some of which have been used in previous research [15]. Although research with alternatives is limited (due to an apparent selection bias for Paro and a limited availability of comparison studies [8, 16]), we previously found evidence that more affordable, less sophisticated devices may offer acceptable alternatives [17], with potential for reproducing the cited benefits of Paro [18].

That said, these reported benefits need to be considered in the context of ethical concerns of robot implementation with older people [19]. In the following, we review some of the relevant literature for the most commonly discussed concerns, including infantilisation, deception, reduced human contact and intrusions on privacy [19–21]. Sparrow and Sparrow [22] assessed the reported capacity of robots to meet older people’s needs, particularly considering social and ethical implications. The authors claim to provide “a much-needed dose of reality” [p:143], suggesting that robots are unable to meet social and emotional needs in almost all aspects of care. They raise the issue of potential for harm, with technological restrictions and potential dangers (eg. trip hazards), removing hopes of robots aiding with personal care, mobility or daily tasks. Potential for harm raises the additional issue of accountability [should harm result from robot implementation [23]. However, the most ethically controversial proposed role for robots appears to be that of companions for older people, the concept of which is sometimes reported as “positively bizarre” [p:308] [21], unethical, and “akin to deception” [p:148] [22].

Regarding deception, some authors feel companion robot benefits rely on delusions as to the real nature of the interaction, described by Sparrow [21] as “sentimentality of a morally deplorable sort” [p:306], with this deceit making robot use misguided and unethical. Sparrow [21] argued robot behaviour is merely imitation: robots do not possess human frailties, and thus cannot ‘understand’ human experience and mortality, rendering them incapable of appropriate, genuine, emotional response [22]. Thus, the extent to which a person feels cared for depends on delusions of robot capabilities. In contrast, Wachsmuth [24] discussed necessity of ‘true’ care for older people, suggesting the illusion of responses to feelings and suffering of the care recipient would suffice, despite a robot’s qualitative experience (without neurophysiological basis for consciousness) not being a ‘true’ caregiver. Sparrow and Sparrow [22] would likely disagree, reporting “the desire to place [robots] in such roles is itself morally reprehensible” [p:154] as robots in roles requiring care, compassion and affection expresses a “gross lack of respect for older persons” [p:156].

Sparrow [21] further suggested that if an older person treats a robot pet as living, thus engaging in the delusion, we have done them a disservice. This appears likely to occur: Robinson et al. [25] noted participants interacted with Paro as a live pet, with some perceiving Paro as having agency despite awareness the device was robotic. The issue of deceit, in particular concerning the distinction between robot and live pet becomes even more problematic with the presence of dementia [26]. Deception is therefore a common ethical concern specific to companion robots that can also be problematic for acceptability among older people’s relatives. Sharkey [19] suggested that, despite a vulnerable older person enjoying robot pets, and perhaps not distinguishing between living and not, relatives may feel they were suffering humiliation and loss of dignity through deception (although it is also possible this tension would ease upon witnessing potential quality of life benefits [27]).

A further ethical issue commonly discussed is reduced human contact. The substantial economic pressures within aged care may result in substitution of human staff with robotic alternatives, which is problematic as human social contact provides significant wellbeing benefits, autonomy and communication opportunities [22]. However, given the regrettably low standard of care provided on occasion by human carers, possibly as a result of high demands including a large workload and low pay [22], there is a well-documented increasing concern that older people can suffer abuse and mistreatment [19]. Dignified treatment by human carers is therefore not a given. In contrast, robots are unable to get angry, abuse an older person or become tired and stressed. Therefore, a small reduction in human contact may be an acceptable compromise for improved quality of care and interaction if robotics could ease strain on human care providers. Support comes from research suggesting reduced carer stress with Paro implementation [11, 28]. Furthermore, robots may mediate social interaction [25], providing a conversation topic between staff, family and older people, and more opportunities to engage socially [19]. Sharkey [19] suggests however, despite solving negatives of human behaviour, robots also lack the true positives; compassion, empathy and understanding. Sparrow and Sparrow [22] argue, due to the crucial role of emotional labour and meaningful conversations for wellbeing, any reduction in human contact would be indefensible.

A further ethical concern is infantilising, an issue also raised for doll therapy, seen by some as congruent with the idea of second childhood, being dispiriting and deficit-based [26, 29]. Infantilisation may damage acceptability for family members, as supported by Robinson et al. [30] who reported that a care resident’s son conveyed their father was not the type to cuddle a soft toy. Another concern is equality of access, as the current cost of companion robots may be prohibitive for people of lower socioeconomic status, who would be denied the potentially therapeutic tool [20, 31].

Whilst the literature is rich with commentary on potential ethical issues, we have been researching real-world robot pet implementation with older people in care homes, and to date, seen limited evidence of ethical concerns amongst older people themselves. We have noted however, occasions where family members have reported such concerns. Family members are key stakeholders in the care of older relatives, and views of relevant stakeholders are fundamental for real-world use [32]. Presenting the views of relevant stakeholders is the core contribution we seek to make with this paper. Successful real-world use of companion robots depends on skilled and careful deployment by relatives and carers [19], thus negative ethical perceptions would likely impair implementation, forming a barrier to adoption [33].

Some previous research has assessed perceptions of older people themselves, including Wu et al. [34], whose results suggested ethical/societal issues presented a potential barrier to robot use, namely privacy and reduced social contact. Pino et al. [32] also conducted a survey and focus group with 25 older people and informal carers, who discussed stigmatisation, privacy issues, dignity, infantilising, replacing human carers, and cost being prohibitively high. Although the exploratory study provided initial insight, with only seven informal carers surveyed, more research is required specific to family member perceptions. A larger sample would additionally allow a comparison between the highlighted concerns to identify the most significant potential barriers. Furthermore, the study involved demonstration of only one robot (RobuLAB 10), with PowerPoint demonstrations of other available socially assistive robots, limiting participant ability to assess robot capabilities [35]. In contrast, we surveyed opinions based on real-world interaction with companion robots, providing informed perceptions with increased validity.

Views of health and social care professionals have also been reported. For example, questionnaire results from 2365 trainee care professionals suggested participants felt companion robots were more beneficial than monitoring or assistive robots, and provided low ratings for maleficence [36]. Nonetheless, research directly surveying ethical perceptions among older people’s family members appears limited. Although much literature debates ethics philosophically, providing a strong overview of potential issues [37], fewer studies specifically assess stakeholder perceptions. Stahl and Coeckelbergh [37] argued that, further to philosophical speculation, we need dialogue and experimentation closer to the context of use. The authors suggest academic reflection on ethics is divorced from the context of practice, with literature mainly addressing what the robot ethics community “think are important ethical issues” [p:154] whilst stakeholder voices remain unheard.

Here, we therefore explore perceptions and prevalence of ethical concerns among younger adults as family members of potential end-users of companion robots, and compare importance of various ethical concerns for this significant stakeholder category, thus contributing to robot ethics understanding for real-world implementation and potential barriers to successful use. This study addresses a timely topic, with real-world and research use of social robot pets increasing, and their use in dementia care being explored, both in the UK and elsewhere [6–18].

## Methods

### Design

This study is a cross-sectional survey with self-completed (with assistance where needed) questionnaires following on from interaction with four companion robots. Previous research relied only on videos and pictures for participants to form opinions [32, 35]. Ethical approval was received from the Science and Engineering ethics committee at the University of Plymouth.

### Procedure and robots

**Fig. 1:**
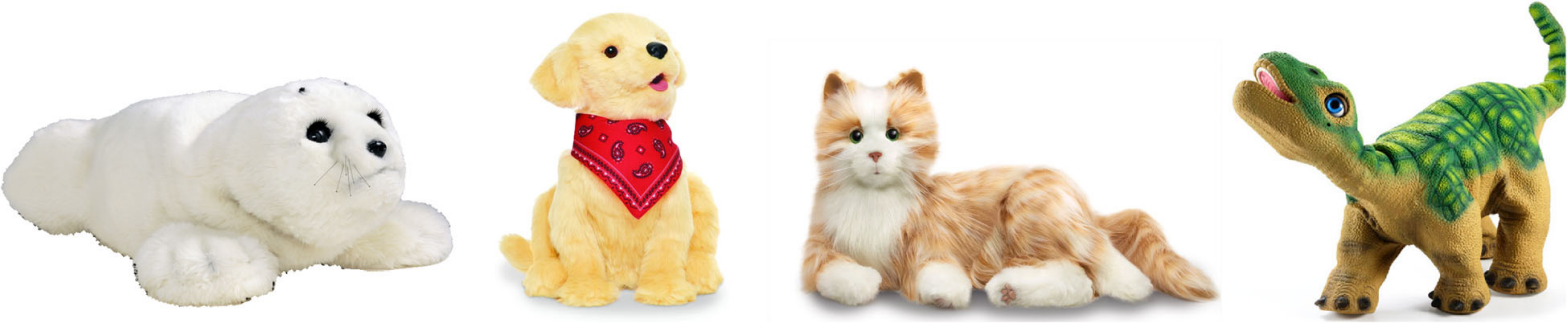
From left, Paro, Joy for All dog, Joy for All cat and Pleo

We hosted an interaction station at a Science Gallery exhibition in November 2018. The overall exhibition comprised 10–15 exhibits exploring the impact of technology on connection (either negative or positive). Visitors to the exhibition were therefore likely to have an interest in issues such as relationships and ethical considerations of technology use in this context. Our station (a room in the Gallery) provided discussions on intimacy for older people, and the potential role of companion robots, and thus served as a good opportunity to survey ethical concerns within context. Participants had the opportunity to interact with four examples of robots and toys for use with older people (Fig. 1).

Participants interacted with devices on the table, or picked up and held devices if they chose. Following interactions, attendees were invited to take part, provided written informed consent, then completed a survey.

### Survey

**Fig. 2:**
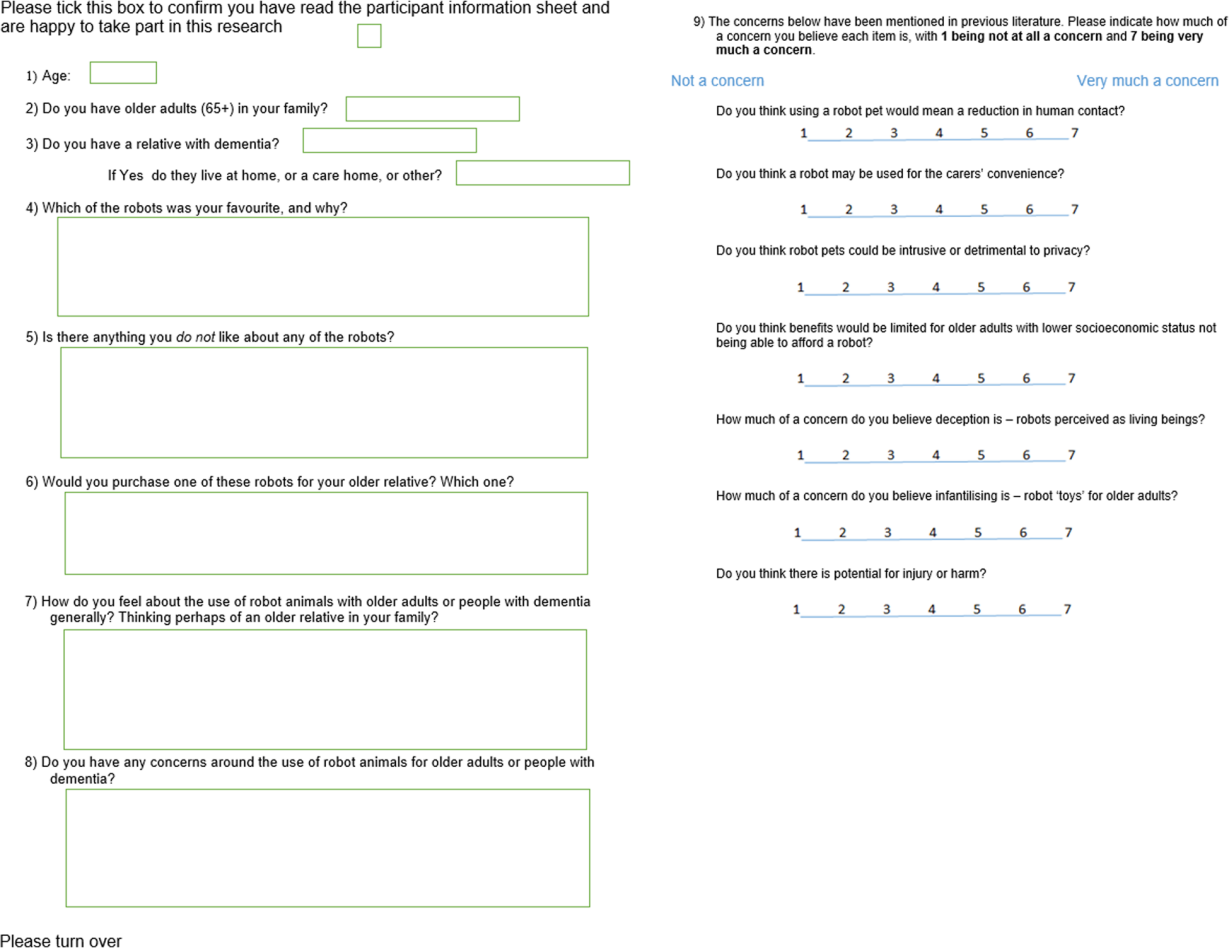
The questionnaire

Based on the literature, we designed a self-completed questionnaire on both sides of one sheet of paper (Fig. 2). The front page asked for participant demographics, which robots they liked and if they might buy one, leading to an open question asking if they had any concerns around the use of robot animals for older people or people with dementia. The back page asked questions based on concerns raised in the literature (reduced human contact, carer’s convenience, privacy, affordability, deception, infantilisation, potential injury) and sought responses using 7-point Likert-type scales questioning the importance of each ethical concern. Each item was scored from 1 (not at all a concern) to 7 (very much a concern).

## Results

Sixty-seven people interacted with the robots and then agreed to complete a questionnaire. They had an average age of 28 years (Range 18–65, SD 10.99). Most (53/67 (79%)) reported having older adult relatives, and 11/67 (16%) had a relative with diagnosed dementia.

Section A of the survey first gained understanding of participant device preferences, likes and dislikes, available in Supplementary File 1. It is worth noting, only one dislike referred to a potential ethical concern (reducing human contact).

Most participants would purchase a device for an older relative (Table 1). Many participants suggested more than one device, and the most popular option was the Joy for All cat. It is also worth noting, that of the 10 participants who reported they would purchase a Paro, four wrote an additional comment such as “if cheaper or more affordable.” Price was also a common reason for participants reporting that they would not buy their relative a device, or a deciding factor on selecting a device other than Paro. This would indicate financial cost is a key deciding factor, with no ethical concerns reported as the reason for not purchasing a device.

**Table 1:** Responses to purchasing a device for an older relative (Q3)

Response	N (%)	Additional			
Yes	39 (58)	**Paro**	**Pleo**	**Cat**	**Dog**
		10	4	14	10
No	21 (31)	**Example Reasons**			
		“Too expensive” “They can decide themselves” “I don’t think they’d like it” “Not into animals” “Not yet” “They have real animals”			
None/Unsure	7 (10)				

Table 2 demonstrates that the majority of participants felt positively when surveyed on general feelings towards companion robots for older people. Within the participants with a mixed response, negative feelings are often justified based on potential benefits. A very small minority provided a completely negative response. Further example evidence can be found in Supplementary File 1.

**Table 2:** Responses to open question on general feelings towards companion robots for older people (Q4)

Response	N (%)	Example Evidence
Positive	44 (66)	“it would be very therapeutic for them” “I think it would be very successful in providing comfort to my relative with dementia, particularly the dog, for nostalgic purposes”
Mixed	10 (15)	“I struggle with the concept of replacing care with robotics but in neurodegenerative diseases such as AZ dementia it can be harder on family members sometimes and if it stimulates/soothes them then maybe” “A good idea, the problem would be making the robot responsive enough without it being too expensive”
Negative	5 (7)	“I would have thought it was a bit ridiculous” “I would be slightly worried of infantilising the person, the person may get upset or see it as a trick”
None	8 (12)	

Most (40/67) reported having no ethical concerns (Table 3). A further five left the box empty, perhaps also indicating a lack of concerns to report, or alternatively reflecting a lack of understanding. This would suggest that prevalence of instinctual ethical concerns is low. The concerns raised by 20 of the 67 participants are summarised in Table 3, demonstrating that deception and reduced human contact were the most prevalent concerns noted by participants upon unprompted questioning of ethical issues. While prevalence was low, the examples do provide some support for the ethical issues reported in previous literature. However, the concerns around battery life, malfunctioning and robustness relate better to the performance of the robot, rather than ethical concerns. Some further examples are available in Supplementary File 1.

**Table 3:** Responses to open question on ethical concerns of companion robot use with older people (%) (Q5)

Response	N (%)			Examples
Concern	20 (30)	**Concern**	**N**	
		Batteries	2	“Emotional distress if the batteries ran out”
		Malfunction	1	“What happens if they malfunction?”
		Human Contact	7	“Might encourage people to be distant from the elderly”
		Robustness	1	“Toughness, can they withstand a fall?”
		Deception	4	“They could become confused as to whether the robot was real or not”
		Privacy	1	“Should not be connected to net (privacy)”
		Danger	2	“Tripping/falling”
		Dignity	2	“They may try to feed or walk them, potential embarrassment”
		Infantilisation	1	“May feel patronised, belittled with a fluffy toy”
No Concern	40 (60)	“No” “None” “No, it seems very safe”		
Unsure	2 (3)	“I don’t know” “Not sure”		
No Response	5 (7)			

Table 4 demonstrates that participants felt the most concerning factor related to equality of access to devices through socioeconomic factors. This concern received the highest mean score, but also the highest median and mode, meaning this issue was most commonly scored as of more concern. The second most concerning issue appears to be robots being used for carer convenience. The least concern was seen for reduced human contact, privacy issues, and potential for injury or harm, all receiving means, modes and medians below the midpoint of 3.5. Infantilising and deception mean scores sit just below the midpoint, whilst the median and mode are just above, demonstrating some concern.

**Table 4:** Potential ethical issues scored on Likert-scales based on level of concern (1 = not at all a concern – 7 = very much a concern)

Potential Issue	Median	Mode	Mean	SD
Socioeconomic Status – Equality of Access	5	6	4.72	1.75
Robots for Carer Convenience	4	5	3.98	1.58
Infantilising	4	4	3.45	1.70
Deception	4	4	3.44	1.61
Reduced Human Contact	3	2	3.06	1.68
Injury or Harm	1	2	2.38	1.67
Privacy	2	1	2.17	1.54

Finally, we acknowledge a possible concern with our participant sample. That is, despite the obvious participant interest in robotics as they attended this exhibition, we recognise 14 out of the 64 participants did not report having an older relative. We therefore analysed (crosstabs and Fisher exact tests) our data from our three key reported outcomes for statistical difference between participants without an older relative, with an older relative and with a relative with dementia. We found no difference between the three groups for the three outcomes we assessed; decision to buy/not buy (Table 1) (.320, *n* = 60, *p* = .925), general perceptions (Table 2) (1.390, *n* = 59, *p* = .618), and ethical concerns (Table 3) (5.897, *n* = 62, *p* = .051). This would suggest the default views of potential future stakeholders is congruent with actual stakeholders.

## Discussion

### Ethical concerns of stakeholders differ from those raised in the literature

We have demonstrated ethical concerns highlighted during philosophical debate of companion robot use [19–23, 26] may differ from those voiced by real-world target groups. The majority of our participants would purchase a companion robot for an elderly relative, suggesting any ethical concerns were not prohibitive to intention to buy. As such, although an awareness of potential issues is evident, they do not appear to weigh strongly enough to act as barriers to successful real-world implementation. In particular, no specific ethical concerns were reported as a reason for not purchasing a device.

The difference we have noted between robot ethics literature and real-world stakeholders is an interesting result: speculative concerns raised in the literature [37] appear mismatched with the priorities of family members within a real-world context. It is of course possible that the lack of significance placed on debated issues by a key stakeholder group may in fact point to a need to increase awareness of these concerns. As such, we have identified a need for further reflections, in the ethics literature, on the implications of a real-world stakeholder group not sharing the same concerns as those raised by the robot ethics community. Whilst stakeholders have demonstrated ethical concerns in previous, mainly qualitative research with small samples [32, 38], re-evaluation may be required in light of these more empirical findings.

### Economic cost is an important factor

Interestingly, economic cost of companion robots presented itself as a continual theme throughout our results, for example as a common reason for not wishing to purchase a device for an older relative further to lack of interest in animals, or limited requirement for such a device. Further support for the central role of the cost barrier comes from participant comments on Paro. Although ten participants suggested they would purchase Paro for a relative, four added the condition “if cheaper.” Financial output is clearly a key deciding factor, and whilst others [39] have demonstrated the cost-effectiveness of Paro as a psychosocial wellbeing activity for older people, the initial expenditure appears prohibitive for family members, a stakeholder group likely to be responsible for purchasing such devices for older relatives. The issue of cost was repeated throughout responses to various questions in our study, including a participant suggesting the challenge faced in companion robot development is “making the robot responsive enough without it being too expensive.” The idea of “responsive enough” is therefore a topic for further exploration [17, 18]. The younger demographic of the sample could also help explain this result, as cost may be less important among a sample of stakeholders already paying for elder care.

### A minority are concerned about reduced human contact and deception

When surveyed with an open question on ethical concerns, most participants reported no concerns. The concerns highlighted by 20/67 (30%) participants however, were congruent with the previous literature. The issues highlighted most often were reduction in human contact, and deception. Companion robots may mediate social contact [25], and reduce care provider burden [11, 28], potentially improving quality of care, therefore further research may be required to directly assess impact on social contact of real-world companion robot implementation, based on both quantity and quality of subsequent human interaction. In the meantime, as suggested by Chiberska [20], we must ensure this technology is applied appropriately. Furthermore, the potential benefits [9–14] make it harder to justify avoiding companion robot use based on ethical concerns [19–23, 26] that do not appear to be a particular concern among family members as real-world stakeholders. It has for example been suggested [27] that family members may justify concerns such as deceit upon witnessing benefits of interaction for their relative. This is supported in our results (Table 2): participants presented conflicted opinions, beginning with an ethical concern and often justifying the issue so long as interactions were beneficial in stimulating or soothing relatives, or eased challenges faced by family members.

The issue of deception is more difficult to mitigate. Whilst real-world companion robot implementers can be mindful of complementing human contact, rather than substituting entirely, ensuring a lack of deception is more difficult when working with individuals with dementia [26]. Older people with dementia may indeed perceive robots as social agents and engage with them as such [18, 21], which is reported within the literature as unethical and problematic [21]. However, with only 4/67 (6%) participants reporting this concern, prevalence is low. This contrasts the specific suggestion that relatives may themselves feel that their family member was suffering humiliation and a loss of dignity through deception [19]. Thus, it does appear that philosophical debate on ethical concerns differs from the priorities of a real-world stakeholder group. As previously noted [37], there is a requirement in the literature to complement the speculative debate with dialogue within the context of use, providing a voice to stakeholders. Our study would suggest family member concerns on deception are unlikely to form a major barrier to real-world use.

Of further interest from the open question on ethical concerns, was that three of the concerns raised (Table 3) related to performance of the device rather than moral ethical concerns. This would suggest these participants did not hold moral concerns around the use of companion robots with older relatives; rather, they wanted to ensure their reliable and successful use.

### Perceived importance of ethical concerns when prompted

The Likert-scales also produced interesting results (Table 4). As we used a 7-point scale, a midpoint would be 3.5. When looking at the means received by each issue, only two were scored above the midpoint of 3.5, and thus suggesting some level of concern: use of robots for carer convenience and inequality of access through socioeconomic status. This provides further support for the impact of high economic cost on the real-world uptake of companion robots, and furthers the argument that the ethical concerns commonly debated [19–23, 26] hold little impact and relevance to family members, as key stakeholders in their older relatives care. Although reduced human contact, privacy issues, infantilisation, deception and potential for injury are commonly debated in robot ethics literatures [19–23, 26], all received means below the midpoint of 3.5, suggesting little prevalence of concern among younger adult family members. Infantilising and deception did receive modes of 4, suggesting some concern, but were still scored of lower concern than carer convenience and equality of access.

### Negative views demonstrate that the suitability of companion robots is not universal

The small number of participants in our survey with negative views towards the robots would suggest these devices are not suitable for everyone, and that there will be incidences of negative response, as seen in previous research [18, 30]. Similar incidences were seen in our survey, such as a participant reporting the idea of companion robots “was a bit ridiculous,” importantly, however, negative views accounted for only 5/67 (7%) responses to the open question on general feelings towards companion robots for older people (Table 2).

### Limitations and strengths

This research has provided important insight into the ethical perceptions of the stakeholder group of younger adult family members, a group that have been shown in previous research to hold impactful opinions towards the real-world use of companion robots [30], and who have been identified as a key stakeholder group to be consulted on ethics [19, 20, 32]. However, a limitation of this study is that there remains a requirement for further dialogue with additional stakeholder groups (older people themselves, care providers, robot designers), to further previous work with small samples and mainly qualitative focus [32, 34] and build a clearer picture of prevalence of ethical concerns within the context of real-world use, as we have. Pino et al. [32] noted that informal carers were less sensitive to privacy concerns than older people with cognitive impairments, who were concerned surveillance applications could damage their privacy. Carers were more positive towards the risk-prevention applications. It is therefore possible that the family members in our research felt more positively about certain ethical aspects than older people would themselves, identifying the importance of further and continuing ethical research with the wider stakeholder groups. Establishing prevalence of ethical concerns is particularly important in the context of ‘real-world’ use, as highly prevalent issues are likely to form barriers to adoption and would signal the requirement for further considerations.

A limitation of our sample is possible distance between our participants and their older relatives, due to the potential participants were not currently directly involved in care of older relatives. It is possible results would differ among a sample of informal carers as stakeholders. Historically, however, family members such as emerging adults (18–25), adolescents and younger children have been neglected from inclusion as stakeholders in older relatives care, despite care involving a whole family system, not only a spouse or older adult child [40]. The lack of similar studies available currently would suggest this neglect is still occurring, highlighting the value of our work and relevance of our participants. Furthermore, younger adults may experience additional impact through the burden experienced by their parents, who may be caring for a grandparent [40]. Expanding our understanding of ‘stakeholder’ could have additional positive implications and acknowledging younger adults as secondary, or perhaps more distant stakeholders could provoke more research into the experiences of this group, and their potential in supporting with the ever-increasing burden of disability associated with the aging population.

We also acknowledge the relatively small sample, but, as noted by others [37], the traditional approach to ethics literature for healthcare robots has mainly involved philosophical reflection, creating a strong requirement for studies that report participant dialogue on ethical concerns acknowledged as limited within the literature. Therefore, our findings are of strong relevance to the social robot and gerontological community in providing interesting data and insight into a previously understudied area. This study also provides the basis for further research, and prompts further ethics studies reporting stakeholder perceptions. An important implication of our work is that it creates further questioning in this area, and should provoke more exploration into a potential misalignment between stakeholders and ethicists, further to investigations into reasoning. Whilst our study does not address the mismatch in full, it does begin the process of endeavour in this area. Future research may also look to develop methodologically, perhaps with video scenarios of specific instances of ethical concerns. Future research might also consider the ethical perceptions of alternative forms of socially assistive robots, such as Pepper [41] that are currently too expensive for widespread use. We chose to focus on robot pets as these devices are currently starting to be deployed across a greater number of situations in real-world implementation, as they are more affordable and accessible.

## Conclusion

We have found interesting differences between the robot ethics community and real-world stakeholders regarding priority concerns for ethical use of companion robots with older adults, which can inform further dialogue in the ethics community. We have further identified a need for ethical literature reflecting on the implications that stakeholders appear not to share the concerns commonly debated in literature. Issues such as infantilisation and deceit appear less relevant to stakeholders of such devices than equality of access due to prohibitively high costs of currently available companion robots. The finding that cost is a primary influential factor is an important outcome of this study, rarely discussed in previous literature, providing an important consideration for robot developers and implementers targeting aged care end-users. A further implication for those working in aged-care is that implementation of such devices is unlikely to encounter many ethical barriers among relatives, despite previously reported concerns.

## Supplementary information

**Additional file 1.** Participant likes and dislikes, and further example evidence.

## Data Availability

Additional data used and/or analysed during the current study are available in the supplementary file.
