# Supplementary material for: Ethical perceptions towards real-world use of companion robots with older people and people with dementia: survey opinions among younger adults
Source: BMC Geriatr. 2020 Jul 14;20:244. doi: 10.1186/s12877-020-01641-5 (PMC7359562; doi:10.1186/s12877-020-01641-5)
Supplement: Supplementary file 1 — Additional file 1. Participant likes and dislikes, and further example evidence. [file 12877_2020_1641_MOESM1_ESM.pdf]

Supplementary File: Participant likes and dislikes, and further example evidence

Participants device preferences and reasons (Q1)

Device	N	Reason
Paro	26	"Reacted to my movements"
		"Pretty eyes, soft"
Pleo	12	"Needy"
		"Interactive and responsive"
Joy for All Dog	20	"Favourite animal"
		"Almost like a real dog"
Joy for All Cat	16	"Fluffiest"
		"Most familiar"

Participant dislikes summary (Q2)

Reason	N	Example
Texture	10	"Fur could be softer"
		"Don't feel real"
		"Too synthetic"
		"Unrealistic"
Internal feel	5	"Hard parts in bodies"
		"Feel plastic under fur"
		"The hard feeling through their fur"
		"Bumpy robot feeling"
Movements	5	"Too still"
		"Random movements, not in response to me"
		"Maybe movements are a little too robotic"

		"Too mechanical"
Size/shape	1	"Cat and dog awkward to hold"
Fear	3	"Creepy"
		"Cat scary"
Cost	1	"Price of seal"
Noises	3	"Dinosaurs noises are annoying"
		"Some noises irritating"
		"Sounds could be better, more sounds"
Ethical concern	1	"I don't really know if they can be used to alleviate loneliness, and whether robots can replace humans in the aspect of intimacy"
Appearance	1	"Dog is one colour, not realistic"
Practical/Maintenance	1	"They can get dirty"

Further evidence of participant responses to open question on general feelings towards companion robots for older people (Q4) – Main text Table 2

Response	%	Example Evidence
Positive	65.76	"It could help them feel more responsible and useful"
		"I really like the idea, it can re-create the feeling of a pet, even if they couldn't look after one"
		"I think anything that could help is a great idea"
		"It is good if it helps them an I know people with dementia can get stressed"
		"Great, why not"
		"Anything that reduces stress is important and beneficial"
		"They are a fun companion to have around and demand very little care"
		"I feel it would be great for them to have some comfort from"
		"I think it would be very therapeutic for them"
		"Great for people who can't have animals"
		"I think it would be very successful providing comfort to my relative with dementia, particularly the dog, for nostalgic purposes"
		"My neighbour has dementia and misses her cat I would love to give her one for comfort"
		"Great idea for people who are alone every day"

Mixed	14.93	"In one way I think it's good as its great that it gives everyone an opportunity to touch but on the other hand I think its sad cause it means that there is no real human contact around them to touch them"<br>"conflicted"<br>"I struggle with the concept of replacing care with robotics but in neurodegenerative diseases such as AZ dementia it can be harder on family members sometimes and if it stimulates/soothes them then maybe"<br>"I wouldn't want people to use it as an excuse not to visit their relatives or look after them but think it's a useful thing as someone can't be with them"<br>"I think it is better to have humans nearby, but if the robots are sufficient to alleviate dementia, I think it is a good idea"<br>"A good idea, the problem would be making the robot responsive enough without it being too expensive"<br>"Maybe it can seem strange, but I think that's a good thing to fight loneliness"<br>"Its sweet and sad, anything that calms them had to be good"<br>"Good concept but I don't know how the old people would react to it"<br>"Good idea, need to lower prices"
Negative	7.46	"Might be more efficient for kids"<br>"I think a real animal would be better"<br>"I would have the thought that it was a bit ridiculous"<br>"I would be slightly worried of infantilising the person, the person may get upset or see it as a trick"
No Response	11.94	

Further evidence of unprompted ethical concerns raised (Q5) – Main text Table 3

Area of Concern	Frequency	Example Evidence
Batteries	2	"There may be emotional distress if the batteries ran out"
Malfunction	1	"What happens if they malfunction?"
Human Contact	7	"I worry about the idea behind it [sic] social care [sic] is about compassion, if we turn that responsibility over to robotics what sort of society will emerge?"<br>"Might encourage people to be distant from the elderly"<br>"People would rely on them too much and not visit"<br>"I may question [sic] the elderly [sic] deserve interactions with real people"<br>"I hope they would never be used to fill an emotional need that a human would give"
Robustness	1	"Toughness, can they withstand a fall?"

Deception	4	"It may confuse them"
		"In cases of bad dementia, they could become confused as to whether the robot was real or not"
		"if the person with dementia thought it was real and found out it wasn't they may be upset."
		"Could become agitated during lucid moments if they feel they were lied to."
Privacy	1	"Should not be connected to net (privacy)"
Danger	2	"Electrical fault if liquid spilt on them"
		"Tripping/falling"
Dignity	2	"They may try to feed or walk them, potential embarrassment"
		"I would hate if they felt patronised or like these were used as a substitute for family"
Infantilisation	1	"May feel patronised belittled with a fluffy toy"
